# Subtyping of Human Papillomavirus-Positive Cervical Cancers Based on the Expression Profiles of 50 Genes

**DOI:** 10.3389/fimmu.2022.801639

**Published:** 2022-01-21

**Authors:** Xiaojun Zhu, Shengwei Li, Jiangti Luo, Xia Ying, Zhi Li, Yuanhe Wang, Mengmeng Zhang, Tianfang Zhang, Peiyue Jiang, Xiaosheng Wang

**Affiliations:** ^1^ Department of Obstetrics, Women’s Hospital, Medical School of Zhejiang University, Hangzhou, China; ^2^ Biomedical Informatics Research Lab, School of Basic Medicine and Clinical Pharmacy, China Pharmaceutical University, Nanjing, China; ^3^ Cancer Genomics Research Center, School of Basic Medicine and Clinical Pharmacy, China Pharmaceutical University, Nanjing, China; ^4^ Big Data Research Institute, China Pharmaceutical University, Nanjing, China; ^5^ Department of Rehabilitation Medicine, First Affiliated Hospital, College of Medicine, Zhejiang University, Hangzhou, China

**Keywords:** human papillomavirus-positive cervical cancer, subtyping, clustering analysis, tumor immune microenvironment, multi-omics

## Abstract

**Background:**

Human papillomavirus-positive (HPV+) cervical cancers are highly heterogeneous in molecular and clinical features. However, the molecular classification of HPV+ cervical cancers remains insufficiently unexplored.

**Methods:**

Based on the expression profiles of 50 genes having the largest expression variations across the HPV+ cervical cancers in the TCGA-CESC dataset, we hierarchically clustered HPV+ cervical cancers to identify new subtypes. We further characterized molecular, phenotypic, and clinical features of these subtypes.

**Results:**

We identified two subtypes of HPV+ cervical cancers, namely HPV+G1 and HPV+G2. We demonstrated that this classification method was reproducible in two validation sets. Compared to HPV+G2, HPV+G1 displayed significantly higher immune infiltration level and stromal content, lower tumor purity, lower stemness scores and intratumor heterogeneity (ITH) scores, higher level of genomic instability, lower DNA methylation level, as well as better disease-free survival prognosis. The multivariate survival analysis suggests that the disease-free survival difference between both subtypes is independent of confounding variables, such as immune signature, stemness, and ITH. Pathway and gene ontology analysis confirmed the more active tumor immune microenvironment in HPV+G1 versus HPV+G2.

**Conclusions:**

HPV+ cervical cancers can be classified into two subtypes based on the expression profiles of the 50 genes with the largest expression variations across the HPV+ cervical cancers. Both subtypes have significantly different molecular, phenotypic, and clinical features. This new subtyping method captures the comprehensive heterogeneity in molecular and clinical characteristics of HPV+ cervical cancers and provides potential clinical implications for the diagnosis and treatment of this disease.

## Introduction

Cervical cancer is the most common gynecological malignancy (1), of which around 90% are squamous cell carcinomas and 10% are adenocarcinomas. Infection by the human papillomavirus (HPV) is the major risk factor for cervical cancer, and about 70% of HPV-related cervical cancer is caused by HPV-16 or HPV-18 (2). Furthermore, previous studies have revealed that HPV-18 infection is an adverse prognostic parameter and that HPV-16 infection has no significant association with survival prognosis in cervical cancer (3). Most of cervical squamous cell carcinoma (CESC) patients are HPV-positive, while about 25% of cervical adenocarcinoma patients are HPV-negative (4). Previous studies have revealed that cervical cancer is highly heterogeneous in clinical and molecular profiles (5). The Cancer Genome Atlas (TCGA) network grouped cervical cancer into four subtypes: HPV clade A9, A7, HPV-negative, and other (5). Besides, TCGA identified three subtypes of cervical cancer, namely keratin-low squamous, keratin-high squamous, and adenocarcinoma-rich by an integrative analysis of copy number, methylation, mRNA and microRNA profiles (5). In addition, TCGA identified three clusters of cervical cancer by reverse phase protein array (RPPA) analysis of 155 samples with 192 antibodies; these clusters included hormone, epithelial-mesenchymal transition (EMT), and PI3K-AKT, of which the EMT cluster showed the worst five-year overall survival outcome (5).

Currently, surgery, chemotherapy and radiotherapy are three major therapeutic options for cervical cancer, although they have limited efficiency for advanced or recurrent cervical cancers (6). Recently, immunotherapies, particularly immune checkpoint inhibitors (ICIs) (7), exhibit efficiency for various solid tumors, such as melanoma, lung cancer, head and neck cancer, kidney cancer, bladder cancer, triple-negative breast cancer, cervical cancer, liver cancer, prostate cancer, and gastrointestinal cancers with mismatch repair deficiency (dMMR). ICIs can induce the regression of certain virus infection-related epithelial malignancies, such as HPV-related cervical (8), head and neck (9), and anal (10) cancers. In fact, the use of ICIs for treating recurrent or metastatic cervical cancers has recently approved by FDA, although less than 20% of cancer patients have an active response to ICIs.

In this study, we identified subtypes of HPV-positive (HPV+) cervical cancers based on gene expression profiles in cervical cancers. Furthermore, we compared molecular and clinical features between the HPV+ cervical cancer subtypes. We also compared molecular features between HPV+ and HPV-negative (HPV-) cervical cancers. This study aimed to explore a new subtyping method for HPV+ cervical cancers and provide potential clinical implications for the diagnosis and treatment of HPV+ cervical cancers.

## Methods

### Datasets

We downloaded three datasets of gene expression profiles in cervical cancer, including TCGA-CESC (5), GSE29570 (11), and GSE39001 (12). The TCGA-CESC dataset was from the genomic data commons (GDC) data portal (https://portal.gdc.cancer.gov/), and GSE30784 and GSE39366 were from the NCBI gene expression omnibus (https://www.ncbi.nlm.nih.gov/geo/). From GDC, we also downloaded the protein expression, somatic mutation, and somatic copy number alteration (CNA) profiles and clinical data in TCGA-CESC. We took TCGA-CESC as the discovery set and performed main analyses in this dataset. The other two datasets were validation sets. These datasets contained only data of human mRNA expression, but not data of the expression of infecting HPV variants. The data on the mRNA expression of HPV alpha-7 and alpha-9 clades were not available in any of these datasets. A summary of these datasets is shown in **Table 1**.

**Table 1:** A summary of the datasets analyzed.

Dataset	# tumors	# HPV+ tumors	# HPV+G1 tumors	# HPV+G2 tumors	# HPV- tumors	Source
TCGA-CESC	303	281	221	60	22	TCGA (https://portal.gdc.cancer.gov/)
GSE29570	62	45	35	10	17	GEO (https://www.ncbi.nlm.nih.gov/geo/)
GSE39001	55	43	25	18	12	GEO (https://www.ncbi.nlm.nih.gov/geo/)

### Clustering Analysis

We first identified 50 genes having the largest expression variations across the HPV+ cervical cancers in TCGA-CESC. Based on the expression profiles of the 50 genes, we performed the hierarchical clustering of the HPV+ cervical cancers in the three datasets, respectively.

### Gene-Set Enrichment Analysis

We quantified the enrichment level of an immune signature or phenotypic feature in a tumor sample by the single-sample gene-set enrichment analysis (ssGSEA) of its marker gene set (13). The ssGSEA calculates the enrichment score of a gene set in a sample based on its expression profiles. The ratios of immunostimulatory/immunosuppressive signatures were the base-2 log-transformed values of the geometric mean expression levels of all marker genes of immunostimulatory signatures divided by those of immunosuppressive signatures. The marker gene sets of immune signatures or phenotypic features are shown in **Supplementary Table S1**.

### Pathway and Gene Ontology (GO) Analysis

We first identified the genes differentially expressed between two classes of samples using the Student’s *t* test with a threshold of adjusted *P* value < 0.05 and fold change of their mean expression levels > 2. By inputting the differentially expressed genes into the GSEA web tool (14), we identified KEGG (15) pathways highly enriched in one class versus another class using a threshold of adjusted *P* value < 0.05. We used the weighted gene co-expression network analysis (WGCNA) (16) to identify gene modules significantly enriched in subtypes. Based on the expression correlations between the gene modules’ hub genes, we identified GO terms having significant correlations with specific trait by WGCNA.

### Calculation of Immune Score, Tumor Purity, Stemness Score and Intratumor Heterogeneity (ITH)

We used the ESTIMATE algorithm (17) to calculate tumor immune score, stromal score, and tumor purity based on gene expression profiles. The immune score, stromal score, and tumor purity indicate the level of tumor-infiltrating lymphocytes, proportion of stromal component, and proportion of tumor cells in a bulk tumor sample. The stemness score was calculated by the ssGSEA of its marker gene set (18) and defined the level of tumor stem cell-like phenotypic feature. We evaluated the ITH level by the DEPTH algorithm (19), which quantifies ITH based on transcriptomic alterations in the tumor.

### Survival Analysis

We used Kaplan–Meier curves to display the survival time difference between cervical cancer subtypes, whose significance was evaluated by the log-rank test. The function “survfit” in the R package “survival” was utilized to perform this analysis. In addition, we performed multivariate survival analysis to investigate the correlation between cervical cancer subtypes and survival prognosis after adjusting for confounding variables, including immune score, stemness score, and ITH score. All these variables are continuous variables. The Cox proportional hazards model was utilized to implement the multivariate survival analysis with the function “coxph” in the R package “survival”.

### Evaluation of Tumor Mutation Burden (TMB) and CNAs

The TMB was defined as the total number of somatic mutations in the tumor. We used GISTIC2 (20) to calculate G-scores for cervical cancer subtypes. The G-score represents the amplitude of the CNA and the frequency of its occurrence across a group of samples (20).

### Class Prediction

We utilized the Random Forest (RF) algorithm (21) to predict cervical cancer subtypes. In the RF, the number of trees was set to 500, and the features included the 50 genes with the largest expression variations across the HPV+ cervical cancers in TCGA-CESC. We reported sensitivity, specificity, and area under the receiver operating characteristic curve (AUC) to evaluate the prediction performance. The R package “randomForest” was utilized to perform the RF algorithm. The R package “PreHPVcc” for predicting HPV+ cervical cancer subtypes is available in the GitHub repository (https://github.com/WangX-Lab/PreHPVcc).

### Statistical Analysis

In class comparisons, we used the Mann–Whitney *U* test or Kruskal–Wallis (K–W) test for not normally distributed data (Shapiro test, *P* < 0.05) and Student’s *t* test or one*-*way analysis *of* variance *(*ANOVA*)* test for normally distributed data. We utilized the chi-square test to analyze contingency tables. We used the Benjamini-Hochberg method (22) to adjust for *P* values in multiple tests. We performed all statistical analyses in the R programming environment (version 4.0.2).

## Results

### Subtyping of HPV+ Cervical Cancers

**Figure 1:**
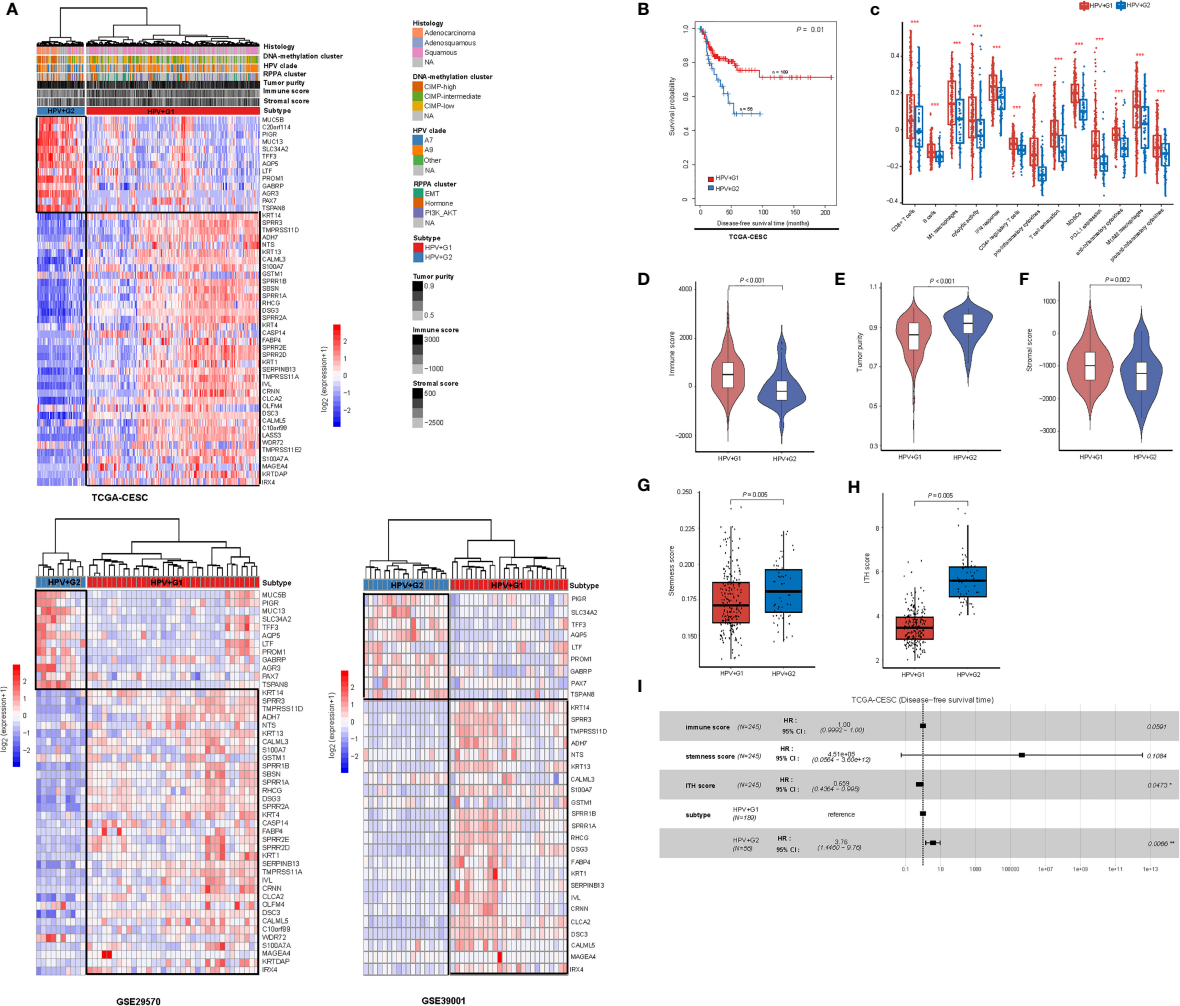
Subtyping of HPV+ cervical cancers based on gene expression profiles. **(A)** Based on the expression levels of the 50 genes having the largest expression variations across the TCGA HPV+ cervical cancers, hierarchical clustering analyses identify two subtypes of HPV+ cervical cancers: HPV+G1 and HPV+G2, consistently in three different datasets. **(B)** HPV+G1 showing significantly higher disease-free survival rate than HPV+G2. The log-rank test *P* value is shown. **(C)** HPV+G1 showing significantly higher enrichment levels of various immune signatures than HPV+G2. The one-tailed Mann–Whitney *U* test or two-tailed Student’s *t* test *P* values are indicated. Comparisons of immune scores **(D)**, tumor purity **(E)**, stromal scores **(F)**, stemness scores **(G)**, and intratumor heterogeneity (ITH) scores **(H)** between HPV+G1 and HPV+G2. The one-tailed Mann–Whitney *U* test *P* values are shown. **(I)** Cox proportional hazards regression analysis showing that the subtype HPV+G2 is a risk factor for disease-free survival prognosis in HPV+ cervical cancers after adjusting for tumor immune signatures, stemness, and ITH. HR, hazard ratio; CI, confidence interval; *P < 0.05, **P < 0.01, ***P < 0.001 (they also apply to the following figures).

We hierarchically clustered HPV+ cervical cancers based on the expression profiles of 50 genes which had the largest expression variations across the HPV+ cervical cancers. We obtained two clear clusters, termed HPV+G1 and HPV+G2 (**Figure 1A**). We confirmed that this classification was reproducible in two other datasets (GSE29570 and GSE39001) (**Figure 1A**). We found that HPV+G1 had a significantly higher disease-free survival (DFS) rate than HPV+G2 (log-rank test, *P* = 0.01) (**Figure 1B**). Interestingly, HPV+G1 showed significantly higher enrichment levels of various immune signatures than HPV+G2, including CD8+ T cells, B cells, M1 macrophages, cytolytic activity, IFN response, CD4+ regulatory T cells, pro-inflammatory cytokines, T cell exhaustion, MDSCs, PD-L1 expression, and anti-inflammatory cytokines (one-tailed Mann–Whitney *U* test, *P* < 0.01) (**Figure 1C**). Moreover, the ratios of immunostimulatory/immunosuppressive signatures (M1/M2 macrophages and pro/anti-inflammatory cytokines) were higher in HPV+G1 than in HPV+G2 (**Figure 1C**). We further used the ESTIMATE algorithm (17) to calculate the immune score representing the tumor immune infiltration level. As expected, immune scores were significantly higher in HPV+G1 than in HPV+G2 (*P* < 0.001) (**Figure 1D**), while tumor purity was significantly lower in HPV+G1 than in HPV+G2 (*P* < 0.001) (**Figure 1E**). These results suggest that HPV+G1 has a more active tumor immune microenvironment than HPV+G2. In addition, we found that stromal scores were significantly higher in HPV+G1 than in HPV+G2 (*P* = 0.002) (**Figure 1F**). HPV+G1 had significantly lower stemness scores than HPV+G2 (*P* = 0.005) (**Figure 1G**). Moreover, HPV+G1 had significantly lower ITH scores than HPV+G2 (*P* = 0.005) (**Figure 1H**).

Because tumor immune signatures, stemness, and ITH are associated with clinical outcomes in cancer (18, 23, 24) and had significantly different scores between the HPV+ cervical cancer subtypes, the survival difference between the subtypes could be impacted by these confounding variables. To explore the possibility, we performed multivariate (immune score, stemness score, ITH score, and subtype) survival analysis with the multivariate Cox proportional hazards model. The result showed that the subtype HPV+G2 remained a significant risk factor (*P* = 0.007; hazard ratio (HR) = 3.76 and its 95% confidence interval (CI) (3):) (**Figure 1I**). It suggests that the DFS difference between both subtypes is independent of these confounding variables.

### Genomic and Epigenomic Profiles of the HPV+ Cervical Cancer Subtypes

**Figure 2:**
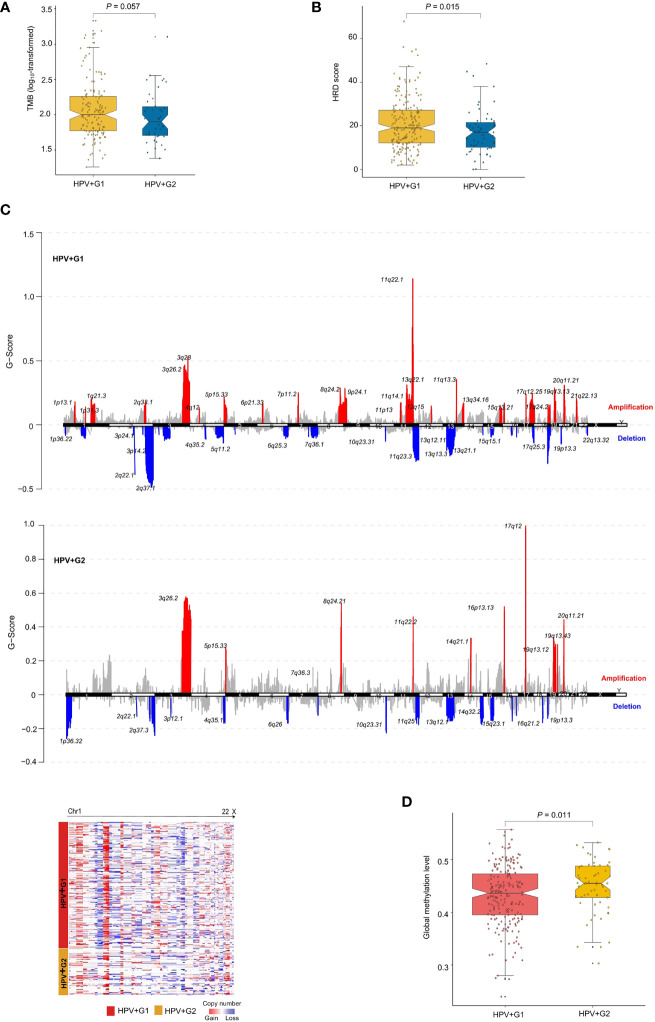
Comparisons of genomic and epigenomic profiles between the HPV+ cervical cancer subtypes. HPV+G1 having higher TMB **(A)**, homologous recombination deficiency (HRD) scores **(B)**, and G-scores of copy number amplifications and deletions **(C)**, and lower global methylation levels **(D)** than HPV+G2. The one-tailed Mann–Whitney *U* test *P* values are shown in **(A, B, D)**. The G-score calculated by GISTIC2 (20) represents the amplitude of the copy number alteration and the frequency of its occurrence across a group of samples.

Genomic instability often leads to increased TMB and CNAs (25). We found that HPV+G1 had higher TMB than HPV+G2 (one-tailed Mann–Whitney *U* test, *P* = 0.057) (**Figure 2A**). Homologous recombination deficiency (HRD) may lead to aneuploidy, namely CNAs (25). We obtained the HRD scores for the TCGA cervical cancers from the publication by Knijnenburg et al., which were the combined scores of HRD loss of heterozygosity, large-scale state transitions, and the number of telomeric allelic imbalances (25). We found that HPV+G1 had significantly higher HRD scores than HPV+G2 (one-tailed Mann–Whitney *U* test, *P* = 0.015) (**Figure 2B**). We found that the G-scores of copy number amplifications and deletions were significantly higher in HPV+G1 than in HPV+G2 cervical cancers (**Figure 2C**). Taken together, these results indicated that HPV+G1 was more genomically instable than HPV+G2.

We compared global methylation levels (26) between both subtypes and found that HPV+G1 had significantly lower global methylation levels than HPV+G2 (one-tailed Mann–Whitney *U* test, *P* = 0.011) (**Figure 2D**). This result conforms with that low methylation levels is associated with increased TMB and CNAs in cancer (26). Strikingly, we found that 5367 genes showed significantly lower methylation levels in HPV+G1 than in HPV+G2 (FDR < 0.05), while there was no any gene showing significantly higher methylation levels in HPV+G1 than in HPV+G2. These results indicate a significant difference in epigenomic profiles between both subtypes.

### Pathways and GO Enriched in the HPV+ Cervical Cancer Subtypes

**Figure 3:**
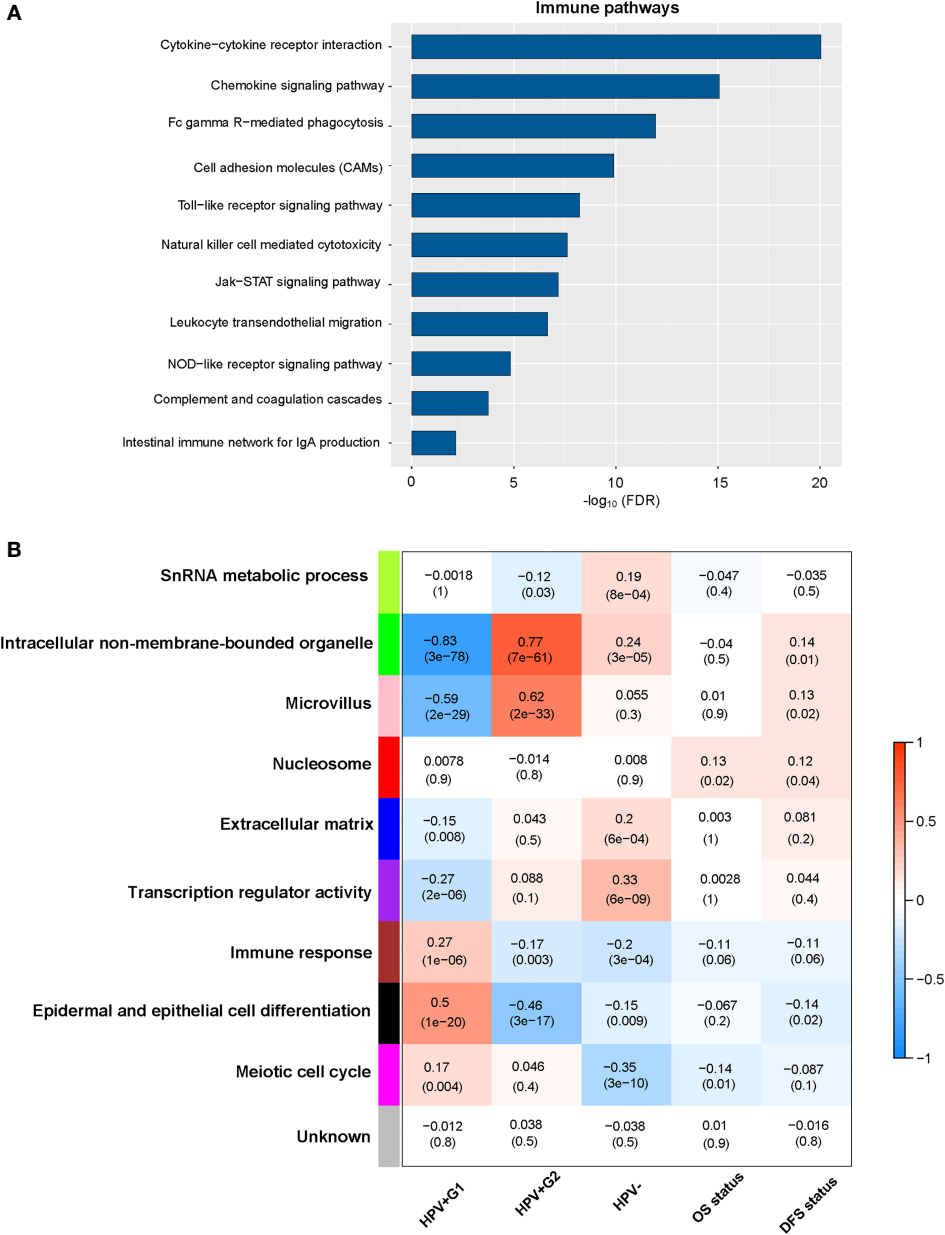
Pathways and gene ontology (GO) enriched in the HPV+ cervical cancer subtypes. **(A)** The immune-related pathways enriched in HPV+G1 versus HPV+G2. **(B)** Nine gene modules and their representative GO terms significantly differentiating cervical cancers by subtype and survival. OS, overall survival; DFS, disease-free survival.

We compared gene expression profiles between HPV+G1 and HPV+G2 and identified significantly upregulated genes in both subtypes. Based on these upregulated genes, we identified KEGG pathways highly enriched in HPV+G1 and HPV+G2, respectively, using the GSEA web tool (14). Many of the pathways especially enriched in HPV+G1 were involved in immune signatures, including cytokine-cytokine receptor interaction, cell adhesion molecules, complement and coagulation cascades, chemokine signaling, Toll-like receptor signaling, Fc gamma R-mediated phagocytosis, leukocyte transendothelial migration, intestinal immune network for IgA production, natural killer cell-mediated cytotoxicity, NOD-like receptor signaling, and Jak-STAT signaling (**Figure 3A**). It confirms the more active tumor immune microenvironment in HPV+G1 versus HPV+G2.

WGCNA (16) identified nine gene modules significantly differentiating cervical cancers by subtype (**Figure 3B**). The representative GO terms for the gene modules upregulated in HPV+G1 while downregulated in HPV+G2 included immune response (in brown) and epidermal and epithelial cell differentiation (in black). In contrast, the representative GO terms for the gene modules upregulated in HPV+G2 while downregulated in HPV+G1 included intracellular non-membrane-bounded organelle (in green) and microvillus (in pink). Again, these results confirm that HPV+G1 has a more active tumor immune microenvironment versus HPV+G2.

### Protein Expression Profiles in the HPV+ Cervical Cancer Subtypes

**Figure 4:**
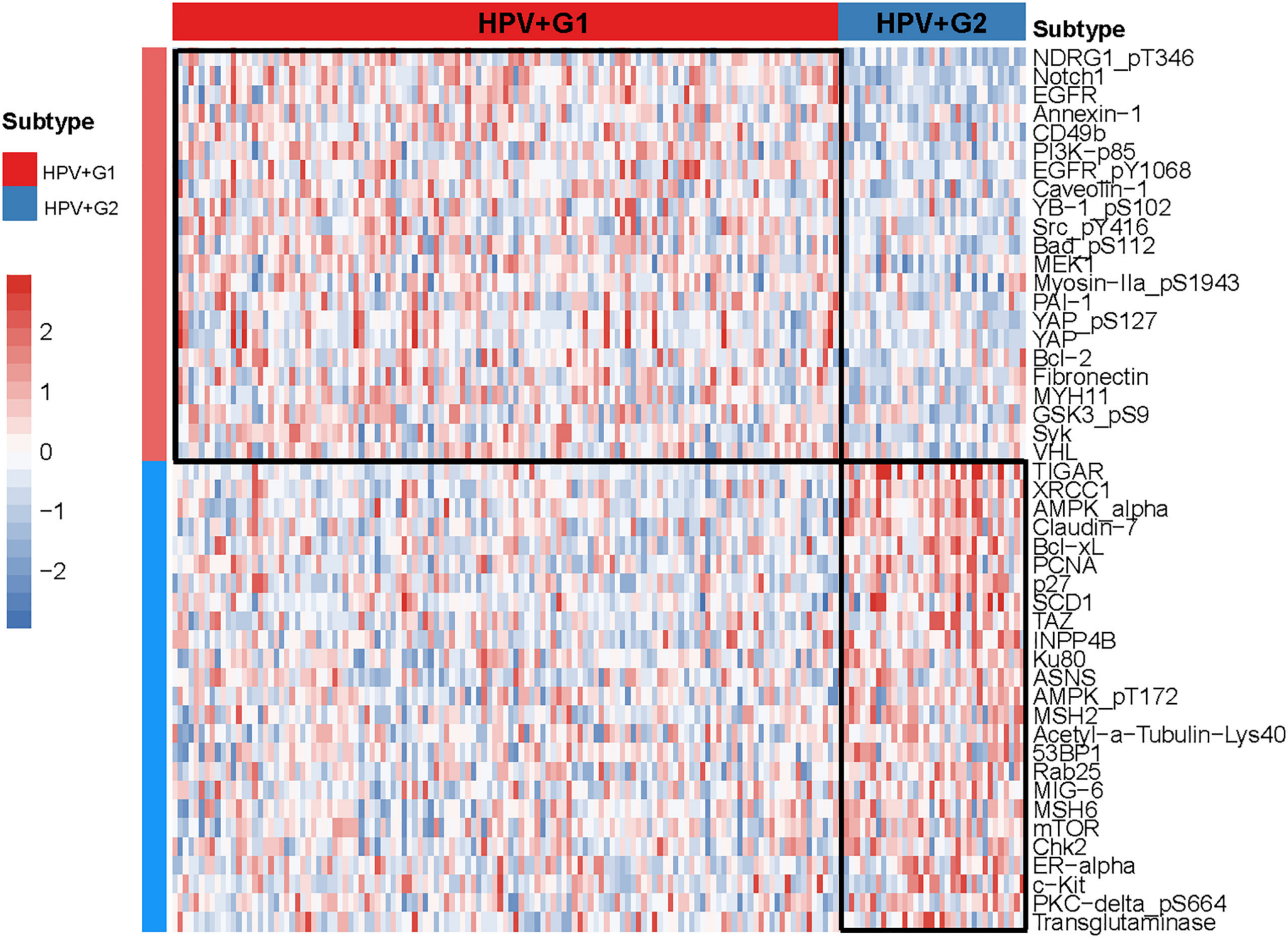
Heatmap showing 22 and 25 proteins upregulated in HPV+G1 and HPV+G2, respectively.

We compared the expression levels of 192 proteins between HPV+G1 and HPV+G2 and identified significantly upregulated proteins in both subtypes (two-tailed Student’s *t* test, *P* < 0.05). We found 22 proteins having significantly higher expression levels in HPV+G1, including NDRG1_pT346, Notch1, EGFR, Annexin-1, CD49b, PI3K-p85, EGFR_pY1068, Caveolin-1, YB-1_pS102, Src_pY416, Bad_pS112, MEK1, Myosin-IIa_pS1943, PAI-1, YAP_pS127, YAP, Bcl-2, Fibronectin, MYH11, GSK3_pS9, Syk, and VHL (**Figure 4**). Among them, Annexin A1, also known as lipocortin I, plays a role in the regulation of innate and adaptive immunity (27). CD49b (cluster of differentiation 49b) is an integrin alpha subunit expressed on various cell types, including T cells and NK cells (28). YAP is involved in the Hippo signaling pathway, which plays a role in tumor immune regulation (29). Bcl-2 is a positive regulator of apoptosis, which is associated with anti-tumor immunity (30). Collectively, these results again confirmed the more active tumor immune microenvironment in HPV+G1 versus HPV+G2.

In contrast, 25 proteins showed significantly higher expression levels in HPV+G2 than in HPV+G1 (**Figure 4**). These proteins included TIGAR, XRCC1, AMPK-α, Claudin-7, Bcl-xL, PCNA, p27, SCD1, TAZ, INPP4B, Ku80, ASNS, AMPK_pT172, MSH2, Acetyl-a-Tubulin-Lys40, 53BP1, Rab25, MIG-6, MSH6, mTOR, Chk2, ER-α, c-Kit, PKC-delta_pS664, and Transglutaminase. Among them, many proteins are involved in DNA repair, including XRCC1, PCNA, Ku80, MSH2, and MSH6, confirming that HPV+G2 was more genomically stable than HPV+G2.

### Comparisons Between the HPV+ Cervical Cancer Subtypes and HPV- Tumors

Although HPV+G2 had lower enrichment levels of immune signatures than HPV+G1, it showed significantly higher enrichment levels of various immune signatures than HPV- tumors, including

NK cells, M1 macrophages, IFN response, CD4+ regulatory T cells, and M2 macrophages (**Supplementary Figure S1A**). Moreover, the ratios of immunostimulatory/immunosuppressive signatures (CD8+ T cells/MDSCs) were significantly higher in HPV+G2 than in HPV- cervical cancers (**Supplementary Figure S1B**). Most of the human leukocyte antigen (HLA) genes, which encode major histocompatibility complex (MHC) proteins and play essential roles in the regulation of the immune system, displayed significantly higher expression levels in HPV+G2 than in HPV- cervical cancers (two-tailed Student’s *t* test, FDR < 0.02; fold change (FC) > 1.5) (**Supplementary Figure S1C**). Immune scores were significantly higher in HPV+G2 than in HPV- cervical cancers (*P* = 0.04) (**Supplementary Figure S1D**). These results support that HPV infection results in a more active tumor immune microenvironment in cervical cancer.

Both ITH and stemness scores followed the pattern: HPV+G1 < HPV+G2 < HPV-, while global methylation levels were higher in HPV+G2 than in both HPV+G1 and HPV- (**Supplementary Figure S1E**). In addition, although TMB and HRD scores were higher in HPV+G1 than in HPV+G2, they were not significantly different between HPV+G2 and HPV- (*P* > 0.2). Furthermore, we did not observe significantly different OS or DFS rate between the HPV+ subtypes and HPV- (log-rank test, *P* > 0.2).

### Prediction of the HPV+ Cervical Cancer Subtypes

**Figure 5:**
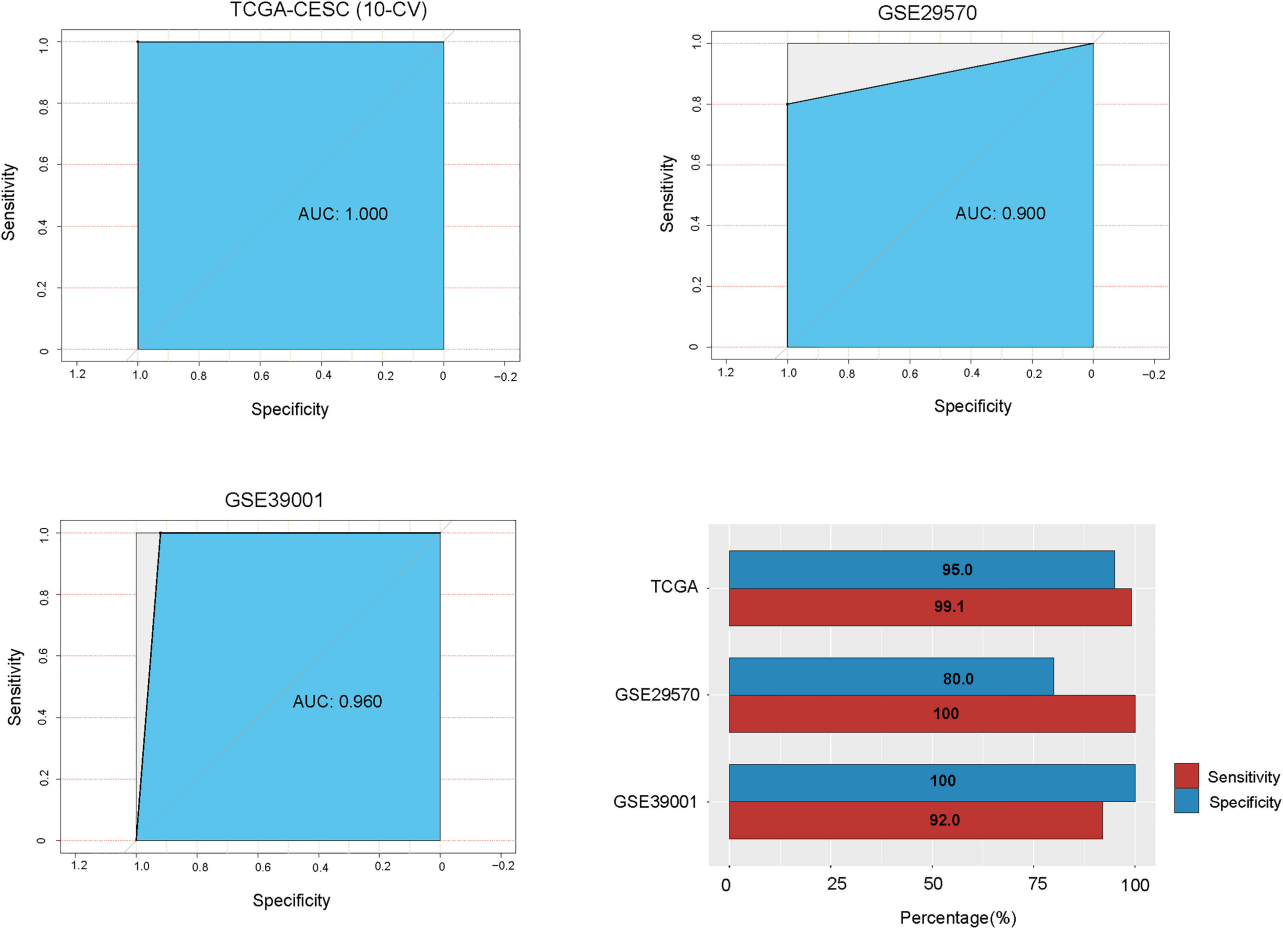
Prediction of the HPV+ cervical cancer subtypes using the 50 genes having the largest expression variations across the TCGA HPV+ cervical cancers by random forest. TCGA-CESC was the training set and GSE29570 and GSE39001 were test sets. The sensitivity, specificity, and AUC are shown. AUC, area under the receiver operating characteristic curve; CV, cross validation.

We used TCGA-CESC as the training set and the other two datasets as test sets. The 10-fold cross-validation (CV) sensitivity, specificity, and AUC in TCGA-CESC were 99.1%, 95.0%, and 100.0%, respectively. The prediction sensitivity, specificity, and AUC in GSE29570 were 100%, 80.0%, and 90.0%, respectively, and in GSE39001 were 92.0%, 100%, and 96.0%, respectively (**Figure 5**). These results suggest that our subtyping method for HPV+ cervical cancers is highly reproducible and predictable. In the prediction model, we found that the 10 features (genes) with the highest importance weights included *DSG3*, *DSC3*, *CLCA2*, *LASS3*, *CALML3*, *SERPINB13*, *IVL*, *PROM1*, *AGR3*, and *TMPRSS11D* (**Table 2**).

**Table 2:** The 10 genes with the highest importance weights in the prediction model.

Symbol	Entrez ID	Full Name	Pathway or biological process*	Importance weight
*DSG3*	1830	Desmoglein 3	Apoptosis; Developmental biology; Keratinization	14.36
*DSC3*	1825	Desmocollin 3	Developmental biology; Keratinization	14.22
*CLCA2*	9635	Chloride channel accessory 2	Activation of cAMP-Dependent PKA;	14.19
			Ion channel transport;	
			Cholera infection	
*CERS3*	204219	Ceramide synthase 3	Sphingolipid metabolism	12.82
*CALML3*	810	Calmodulin like 3	B cell receptor signaling;	12.23
			MAPK-Erk pathway	
*SERPINB13*	5275	Serpin family B member 13	regulation of keratinocyte differentiation	10.63
*IVL*	3713	Involucrin	Keratinization;	8.12
			G-beta gamma signaling;	
			Developmental biology; Corticotropin-releasing hormone signaling	
*PROM1*	8842	Prominin 1	Wnt/Hedgehog/Notch; Embryonic and induced pluripotent stem cells and lineage-specific markers;	8.07
			Neural stem cells and lineage-specific markers	
*AGR3*	155465	Anterior gradient 3, protein disulphide isomerase family member	Estrogen receptor signaling	7.60
*TMPRSS11D*	9407	Transmembrane serine protease 11D	Regulation of viruses into host cells	6.89

*The pathways or biological processes the genes involved in were obtained from the GeneCards (https://www.genecards.org/) and NCBI (https://www.ncbi.nlm.nih.gov/gene/).

### Overlapping Between the HPV+ Subtypes and Other Subtypes of Cervical Cancer

**Figure 6:**
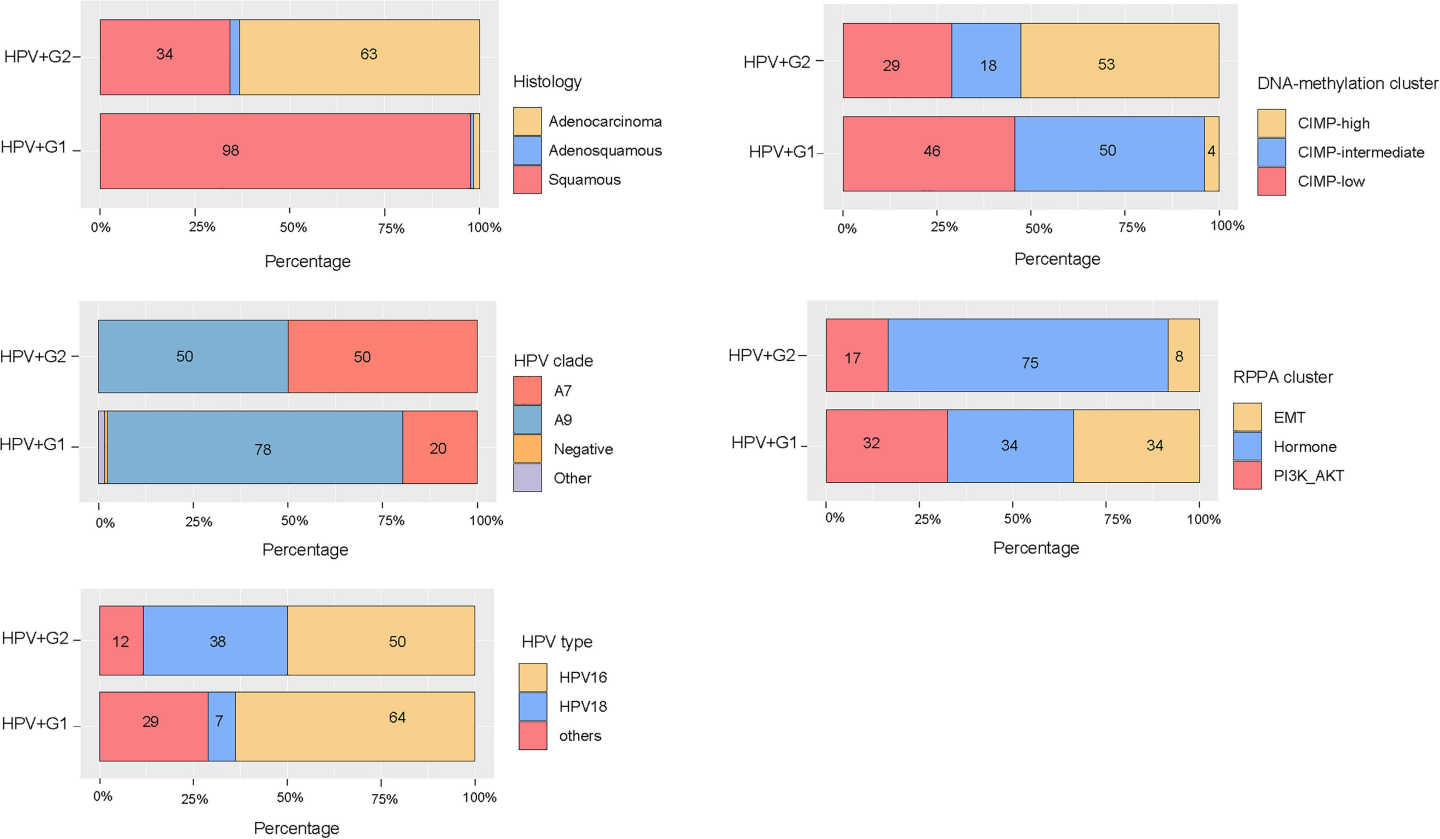
Overlapping between the HPV+ subtypes and other subtypes of cervical cancer.

We investigated the relationship between our subtyping method and other cervical cancer subtyping methods (5). We found that squamous cell carcinomas constituted 98% of HPV+G1 tumors versus 34% of HPV+G2 tumors (chi-square test, *P* < 0.001) (**Figure 6**). In contrast, adenocarcinomas constituted 63% of HPV+G2 tumors versus 2% of HPV+G1 tumors. The TCGA network classified cervical cancers into three subtypes by unsupervised clustering of variable DNA-methylation probes (5). The three subtypes included: CpG island hypermethylated (CIMP-high), CIMP-intermediate, and CIMP-low. We found that 4% of HPV+G1 tumors were CIMP-high, compared to 53% of HPV+G2 tumors being CIMP-high, and that 46% of HPV+G1 tumors were CIMP-low versus 29% of HPV+G2 tumors being CIMP-low (*P* < 0.001) (**Figure 6**). It is consistent with the lower overall DNA methylation level in HPV+G1 relative to HPV+G2. In addition, we found 20% of HPV+G1 tumors being Clade A7 versus 50% of HPV+G2 tumors and 78% of HPV+G1 tumors being Clade A9 versus 50% of HPV+G2 tumors (*P* = 0.002) (**Figure 6**). The different distribution of HPV clades between HPV+G1 and HPV+G2 indicates a better prognosis in HPV+G1 versus HPV+G2 since HPV clade A7 cervical cancers are more aggressive than clade A9 cancer (31). RPPA-based clustering identified three clusters: hormone, EMT, and PI3K-AKT (5). We found that 34% of HPV+G1 tumors were in the EMT cluster versus 8% of HPV+G2 tumors (*P* = 0.002) (**Figure 6**). It is justified since EMT represents a stromal signature and HPV+G1 has higher stromal scores than HPV+G2. In addition, 32% of HPV+G1 tumors were in the PI3K-AKT cluster versus 17% of HPV+G2 tumors, and 34% of HPV+G1 tumors were in the hormone cluster versus 75% of HPV+G2 tumors. It is consistent with previous results that PI3K-p85 was more abundant in HPV+G1 while ER-α was more abundant in HPV+G2. Furthermore, 37% of HPV+G1 tumors were HPV-16 positive versus 32% of HPV+G2 tumors, and 5% of HPV+G1 tumors were HPV-18 positive versus 25% of HPV+G2 tumors. It indicates that a significantly higher proportion of HPV+G2 tumors are HPV-18 positive compared to HPV+G1 tumors (*P* < 0.001). This result supports that HPV-18 infection is an adverse prognostic factor in cervical cancer (3), while HPV-16 infection is a positive prognostic factor in cervical cancer (32).

## Discussion

Based on the expression profiles of the 50 genes with the largest expression variations across the HPV+ cervical cancers in TCGA-CESC, we identified two subtypes of HPV+ cervical cancers, namely HPV+G1 and HPV+G2. We demonstrated that this classification method was reproducible in two validation sets. Compared to HPV+G2, HPV+G1 displayed significantly higher immune infiltration level and stromal content, lower tumor purity, lower stemness scores and ITH scores, higher level of genomic instability, lower DNA methylation level, as well as more favorable prognosis. The multivariate survival analysis suggests that the survival difference between both subtypes is independent of confounding variables, such as immune signature, stemness, and ITH. It is interesting to observe that HPV+G1 has a better DFS prognosis than HPV+G2, while HPV+G1 is more genomically instable compared to HPV+G2. Genomic instability is a common characteristic of cancers that drives cancer development (33). Nevertheless, increased TMB resulting from genomic instability may have generated more neoantigens that were shown to promote antitumor immune response (34). This could explain why patients in group HPV+G1 have a more favorable prognosis than in HPV+G2, even though the former display a higher level of genomic instability than the latter (35).

Among the 50 genes for clustering analysis, 37 showed higher expression levels in HPV+G1 versus HPV+G2. These genes included four members of the keratin gene family: *KRT1*, *KRT4*, *KRT13*, and *KRT14*. It supports previous findings that keratin expression plays a role in cervical cancer classification (36). The 37 genes also included two genes encoding calmodulin-like proteins: *CALML3* and *CALML5*. *CALML5* has been identified as a tumor suppressor gene in squamous cell carcinoma of uterine cervix (37). It consistent with that *CALML5* is upregulated in HPV+G1 which has a more favorable prognosis versus HPV+G2. *CALML3* and *CALML5* are involved in the Ras, Rap1, calcium, neurotrophin, and estrogen signaling pathways, which plays important roles in cervical cancer development (38–42). In addition, the 37 genes included six members of the small proline-rich protein gene family: *SPRR1A*, *SPRR1B*, *SPRR2A*, *SPRR2D*, *SPRR2E*, and *SPRR3*. Previous studies have revealed that many small proline-rich protein genes were downregulated in cervical cancer relative to normal cervical tissue (43, 44), suggesting their tumor suppressor roles. It conforms to the better prognosis in HPV+G1 versus HPV+G2. 13 of the 50 genes for clustering analysis were more highly expressed in HPV+G2 than in HPV+G1, including *MUC5B*, *BPIFB1*, *PIGR*, *MUC13*, *SLC34A2*, *TFF3*, *AQP5*, *LTF*, *PROM1*, *GABRP*, *AGR3*, *PAX7*, and *TSPAN8*. Among them, *MUC5B* and *MUC13* belong to the mucin gene family, which plays oncogenic roles in various cancers (45, 46). Previous studies also showed that mucin genes were associated with subtyping of cervical cancer (5). PAX7 is a member of the paired box (PAX) family of transcription factors and is oncogenic in a variety of cancers, including cervical cancer (47). Again, *PAX7* upregulation in HPV+G2 relative to HPV+G1 is in line with the better prognosis in HPV+G1 versus HPV+G2. Interestingly, 47 of the 50 genes for clustering analysis were differentially expressed between HPV-16 positive HPV+G1 and HPV-18 positive HPV+G2 tumors (FDR < 0.01, FC > 2). The 47 genes included 34 genes which were more highly expressed in HPV-16 positive HPV+G1 versus HPV-18 positive HPV+G2 tumors and were also more highly expressed in HPV+G1 versus HPV+G2 tumors. In contrast, the other 13 genes were more highly expressed in HPV-18 positive HPV+G2 versus HPV-16 positive HPV+G1 tumors and were also more highly expressed in HPV+G2 versus HPV+G1 tumors. These data suggest that the HPV genotype could exert a significant effect on the expression pattern of most of the 5o genes since the HPV-18 genotype has a significantly different distribution between HPV+G1 and HPV+G2. In fact, previous studies have shown that HPV infection is able to cause global gene expression changes at the precancerous and cancerous stages of cervical cancer (48–50). For example, HPV-16 and HPV-18 E6 oncoproteins promote the deregulation of tumor suppressor genes, such as *TP53* and *RB1*, to induce the expression changes of their target genes (51–53). Interestingly, some of the 50 genes, such as *DSG3* and *CLCA2*, have been identified as targets of p53 (54, 55).

The TCGA network also performed mRNA clustering analysis to identify cervical cancer subtypes using the uncentered correlation and centroid linkage method (5). This method discovered three cervical cancer subtypes: C1, C2, and C3. We found that 95% of HPV+G2 tumors belonged to C1 and that 72% and 25% of HPV+G1 tumors belonged to C2 and C3, respectively. It indicates that HPV+G2 is nearly equivalent to C1 and that HPV+G1 is the combination of C2 and C3. However, survival analysis showed that there was no significant difference in DFS among C1, C2, and C3, compared to significant difference in DFS between HPV+G1 and HPV+G2. It suggests that our mRNA-based subtyping method is more reasonable than that method in terms of the prognostic relevance.

## Conclusions

HPV+ cervical cancers can be classified into two subtypes based on the expression profiles of the 50 genes with the largest expression variations across the HPV+ cervical cancers. Both subtypes have significantly different immune and stromal microenvironment, tumor purity, stemness, ITH, genomic instability, DNA methylation level, as well as survival prognosis. This new subtyping method captures the comprehensive heterogeneity in molecular and clinical characteristics of HPV+ cervical cancers and provides potential clinical implications for the management of this disease.

## Data Availability Statement

Publicly available datasets were analyzed in this study. This data can be found here: TCGA-CESC (https://portal.gdc.cancer.gov) and the NCBI gene expression omnibus (https://www.ncbi.nlm.nih.gov/geo/) under the accession numbers GSE30784 and GSE39366.

## Author Contributions

XZ and SL, software, validation, formal analysis, investigation, data curation, and visualization. JL, investigation and data curation. XY, formal analysis and investigation. ZL, YW, MZ, and TZ, formal analysis. PJ, investigation, resources, supervision, funding acquisition. XW, conceptualization, methodology, resources, investigation, writing - original draft, writing - review and editing, supervision, and project administration. All authors contributed to the article and approved the submitted version.

## Funding

This work was supported by the National Natural Science Foundation of China (grant number 81902625).

## Conflict of Interest

The authors declare that the research was conducted in the absence of any commercial or financial relationships that could be construed as a potential conflict of interest.

## Publisher’s Note

All claims expressed in this article are solely those of the authors and do not necessarily represent those of their affiliated organizations, or those of the publisher, the editors and the reviewers. Any product that may be evaluated in this article, or claim that may be made by its manufacturer, is not guaranteed or endorsed by the publisher.

## Supplementary Material

Supplementary Figure 1 Comparisons between the HPV+ cervical cancer subtypes and HPV- tumors. Higher enrichment levels of immune signatures **(A)**, ratios of immunostimulatory/immunosuppressive signatures (CD8+ T cells/MDSCs) **(B)**, expression levels of human leukocyte antigen (HLA) genes **(C)**, and immune scores **(D)** in HPV+G2 than in HPV- cervical cancers. **(E)** Comparisons of ITH scores, stemness scores, and global methylation levels among cervical cancer subtypes. The K–W or one-way ANOVA test **(A, C)**, two-tailed Student’s *t* test **(B)**, and one-tailed Mann–Whitney *U* test **(D, E)** *P* values are indicated. * *P* < 0.05, ** *P* < 0.01, *** *P* < 0.001.

## Abbreviations

HPV, human papillomavirus; HPV+, Human papillomavirus-positive; HPV-, HPV-negative; CESC, cervical squamous cell carcinoma; EMT, epithelial-mesenchymal transition; dMMR, mismatch repair deficiency; TMB, tumor mutation burden; CNA, copy number alteration; ITH, intratumor heterogeneity; HRD, Homologous recombination deficiency; PR, progesterone receptor; ICIs, immune checkpoint inhibitors; RF, Random Forest; DFS, disease-free survival; HR, hazard ratio; HLA, human leukocyte antigen; MHC, major histocompatibility complex; CV, cross-validation; CIMP, CpG island hypermethylated; WGCNA, weighted gene co-expression network analysis.
