# Supplementary figures and images for: Subtyping of Human Papillomavirus-Positive Cervical Cancers Based on the Expression Profiles of 50 Genes
Source: Front Immunol. 2022 Jan 21;13:801639. doi: 10.3389/fimmu.2022.801639 (PMC8814347; doi:10.3389/fimmu.2022.801639)

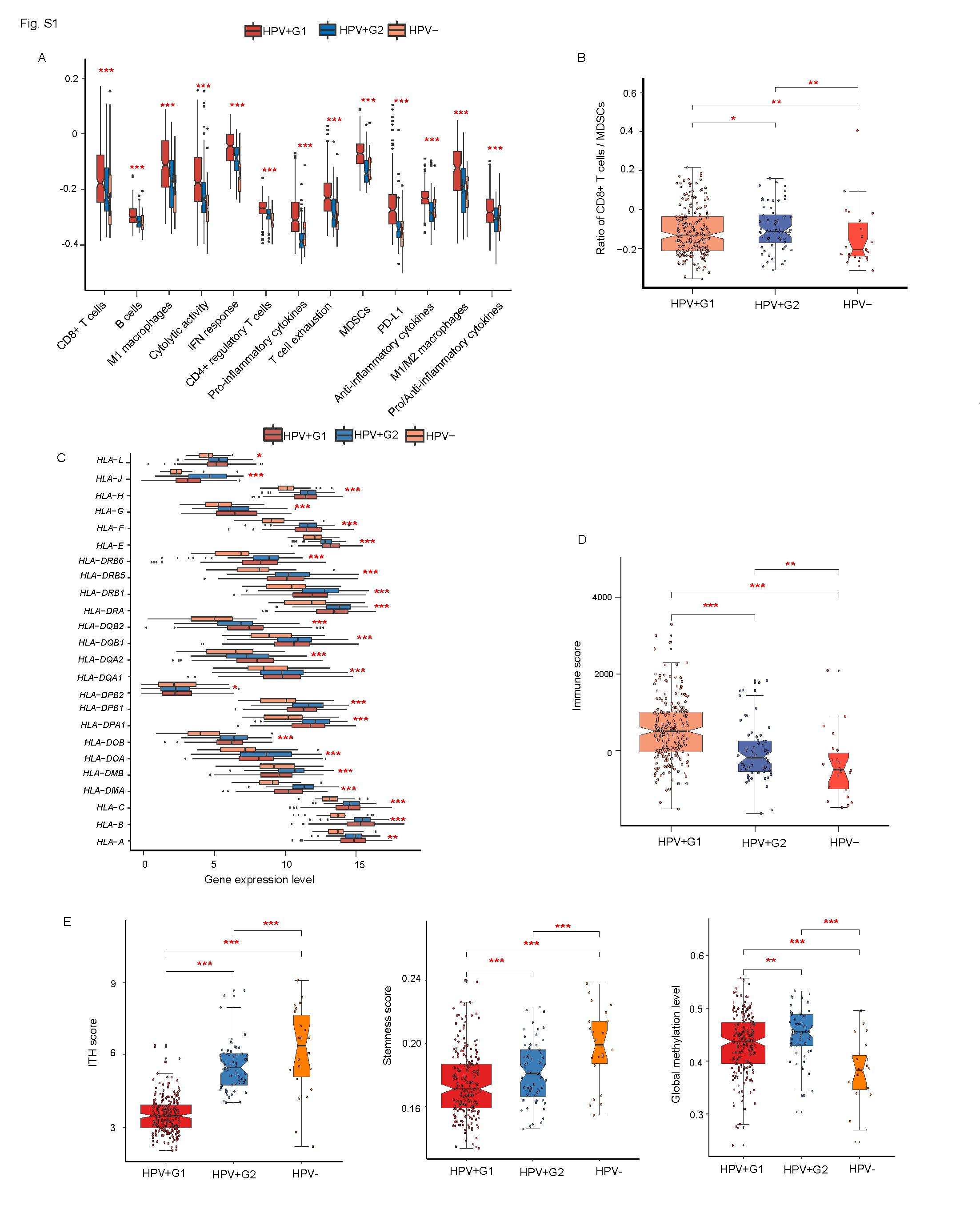

Supplement: Supplementary Figure 1 — Comparisons between the HPV+ cervical cancer subtypes and HPV- tumors. Higher enrichment levels of immune signatures (A), ratios of immunostimulatory/immunosuppressive signatures (CD8+ T cells/MDSCs) (B), expression levels of human leukocyte antigen (HLA) genes (C), and immune scores (D) in HPV+G2 than in HPV- cervical cancers. (E) Comparisons of ITH scores, stemness scores, and global methylation levels among cervical cancer subtypes. The K–W or one-way ANOVA test (A, C), two-tailed Student’s t test (B), and one-tailed Mann–Whitney U test (D, E) P values are indicated. * P < 0.05, ** P < 0.01, *** P < 0.001. [file Image_1.tif]
